# How long should the COVID-19 lockdown continue?

**DOI:** 10.1371/journal.pone.0243413

**Published:** 2020-12-02

**Authors:** Jonathan Caulkins, Dieter Grass, Gustav Feichtinger, Richard Hartl, Peter M. Kort, Alexia Prskawetz, Andrea Seidl, Stefan Wrzaczek

**Affiliations:** 1 Heinz College, Carnegie Mellon University, Pittsburgh, Pennsylvania, United States of America; 2 International Institute for Applied Systems Analysis (IIASA), Laxenburg, Austria; 3 Research Unit Operations Research and Control Systems (ORCOS), Institute of Statistics and Mathematical Methods in Economics, Vienna University of Technology, Vienna, Austria; 4 Wittgenstein Centre for Demography and Global Human Capital (Univ. Vienna, IIASA, VID/OAW), Vienna, Austria; 5 Department of Business Decisions and Analytics, University of Vienna, Vienna, Austria; 6 Tilburg School of Economics and Management, Tilburg University, Tilburg, Netherlands; 7 Department of Economics, University of Antwerp, Antwerp, Belgium; 8 Research Unit Economics (ECON), Institute of Statistics and Mathematical Methods in Economics, Vienna University of Technology, Vienna, Austria; Nanyang Technological University, SINGAPORE

## Abstract

Nations struggled to decide when and how to end COVID-19 inspired lockdowns, with sharply divergent views between those arguing for a resumption of economic activity and those arguing for continuing the lockdown in some form. We examine the choice between continuing or ending a full lockdown within a simple optimal control model that encompasses both health and economic outcomes, and pays particular attention to when need for care exceeds hospital capacity. The model shows that very different strategies can perform similarly well and even both be optimal for the same relative valuation on work and life because of the presence of a so-called Skiba threshold. Qualitatively the alternate strategies correspond to trying essentially to eradicate the virus or merely to flatten the curve so fewer people urgently need healthcare when hospitals are already filled to capacity.

## Introduction

The novel SARS-CoV-2 virus has literally swept around the globe. A prominent countermeasure has been to “lock down” non-essential parts of the economy to reduce contagious spread through social interaction. Lockdowns have succeeded in “flattening the curve” but at a high price; job losses in many places are the highest ever recorded, and there have been effects on stock markets not seen with prior epidemics (see [1]). That raises the question of when such measures should be relaxed. Too soon and the epidemic will bounce back; too late and there is needless economic hardship.

This paper analyzes a simple optimal control model of that difficult balancing. It supplements an SLIR epidemic model with controls that temporarily remove a proportion of the population from both social interaction and the workforce. The objective function encompasses both health and economic considerations. Specifically, it recognizes benefits from people being free to work and costs when infected people need hospitalization, with an extra penalty when the number needing hospitalization exceeds hospital capacity.

The modeling of policy here is very simple, involving just picking the beginning and ending times of the lockdown, denoted by *τ*_1_ and *τ*_2_, respectively. In reality there are various ways of partially easing a lockdown without ending it completely, so the policy choice modeled here is stylized.

Since most jurisdictions had already begun their lockdown, we initially planned to optimize only over *τ*_2_, for varying values of *τ*_1_. The results were surprising.

As might be expected, the optimal duration of the lockdown depends sharply on when it began, but not always in the expected way. Whereas one might have thought that starting too late would require keeping the lockdown in place longer, we find that starting later can sometimes make it optimal to end the lockdown sooner.

To understand the origins of this behavior, we formulate the model more generally to optimize over both *τ*_1_ and *τ*_2_. Doing so reveals alternative optimal strategies that differ only modestly concerning timing of lockdown initiation, but which differ markedly in terms of health and economic outcomes. Later starts lead to health outcomes that are just enough worse to offset for the earlier re-opening. Remarkable is that both optimal solutions prevail under the same prioritization regarding health versus economic growth. Alternative optimal solution trajectories in dynamic models have variously been called Skiba, Sethi-Skiba, DNS, and DNSS points recognizing the contributions of various pioneers in the field [2]. The one here appears to be of a new type. Rather than emerging from interactions between the state and control variables, here path dependence is generated by an interaction between timing and duration.

The literature on COVID-19 models is exploding almost as fast as the virus itself, and some papers do model the balancing of health and economic interests (see [3, 4] and [5] for a careful evaluation). [6] depart from the SIR dynamics and have economic activity as a control variable. Increasing this variable on the one hand raises output, but on the other hand it feeds the number of infections, raising the burden on the health care system. Calibrating their model based on the U.S. situation, they suggest a lockdown of 50 days in which economic activity is reduced by two-thirds.

[7] also employ the SIR dynamics, but their control variable is the fraction of the population going into lockdown. They particularly include the effect of testing. Conditional on the possibility of testing and after parametrizing their model using data from the World Health Organization (WHO), they find that it is optimal to start a lockdown one week after the outbreak, and after one month it will be gradually withdrawn. Absence of testing increases the economic costs of the lockdown and shortens its optimal duration.

[8] investigate a heterogeneous SIR model distinguishing between the “young”, the “middle-aged” and the “old”. They find that it is especially beneficial to have stricter lockdown policies on the oldest group. [9] consider a stochastic version of the SIR model. They use a continuous-time Markov chain model to study the value and optimal timing of two (sequential) options: the option to intervene and, after intervention has started, the option to end it.

We, like they, recognize that locking down the population in order to reduce transmission also reduces employment. Our primary additional innovation on the modeling side is distinguishing between the health consequences of infection when proper care is received from those when the healthcare system’s capacity to render appropriate care has been exceeded. The number of deaths is not simply proportional to the number of infections; there is an extra penalty for infections that happen when hospitals are overwhelmed. Our primary methodological contribution is exploration of threshold effects when the optimal solution “tips” from one strategy to another.

Like us, [10] extend the SIR framework by including a state for individuals exposed to the virus but not symptomatic, and they also consider limited capacity of the health system. Their main result is that testing generates considerable welfare gains.

Where the just mentioned papers, including ours, formulate planner problems, [11] consider a competitive equilibrium in which a consumption tax is used to slowdown economic activity and epidemic diffusion. [12] extend [11] by distinguishing goods by the degree to which they can be consumed at home rather than in a social (and thus possibly contagious) context.

The model analyzed here is quite simple. It does not, for example, distinguish between young and old or consider movement between jurisdictions. So although we choose parameters that produce epidemic curves broadly consistent with those of other models, we stress the qualitative conclusions only.

The paper is organized as follows. The next section presents the model and elaborates the choice of the parameter values. The numerical results and their implications are discussed in the section ‘Results’. The section ‘Conclusions’ concludes.

## The model

### SLIR model

The backbone of the model is an open-population SLIR model [13] with a birth rate *ν* and extra mortality for individuals who are infected (*μ*_*I*_) above and beyond that for those who are susceptible or recovered (*μ*). We introduce the birth and background death rate parameters for completeness. For the qualitative results these are of no importance since the time horizon for lockdowns is relatively short. Given the state variables:

*S*(*t*): Number of susceptible individuals at time *t*
*L*(*t*): Number of latent (asymptomatic and pre-symptomatic) individuals at time *t*
*I*(*t*): Number of infected individuals who are showing symptoms at time *t*
*R*(*t*): Number of recovered individuals at time *t*,

the SLIR state equations are:

$$\begin{array}{cc} & \dot{S}\left(t\right)=\nu N\left(t\right)-\beta \frac{S(t)(I(t)+fL(t))}{N(t)}-\mu S\left(t\right) \end{array}$$ (1a)

$$\begin{array}{cc} & \dot{L}\left(t\right)=\beta \frac{S(t)(I(t)+fL(t))}{N(t)}-\left(\mu +\varphi \right)L\left(t\right) \end{array}$$ (1b)

$$\begin{array}{cc} & \dot{I}\left(t\right)=\omega \varphi L\left(t\right)-\left(\alpha +\mu +\mu_{I}\right)I\left(t\right) \end{array}$$ (1c)

$$\begin{array}{cc} & \dot{R}\left(t\right)=\left(1-\omega \right)\varphi L\left(t\right)+\alpha I\left(t\right)-\mu R\left(t\right) \end{array}$$ (1d)

$$\begin{array}{c} \beta ≔R_{\text{eff}}\left(t,\tau_{1},\tau_{2}\right)\alpha \end{array}$$ (1e)

$$\begin{array}{c} N(t)≔S(t)+L(t)+I(t)+R(t). \end{array}$$

### Lockdown’s effect on epidemic spread

The lockdown directly affects the rate of social interaction, *β*, but the effective reproduction number *R*_eff_(*t*, *τ*_1_, *τ*_2_) is more readily interpretable, so we describe the lockdown phases in terms of effects on *R*_eff_(*t*, *τ*_1_, *τ*_2_) and adjust *β* accordingly (for a formal derivation of the basic reproduction number *R*_0_ we refer to Appendix 2).

We distinguish three periods: before, during and after the lockdown, denoted by the subscripts 1, 2, and 3, respectively. The decision maker gets to choose the times, *τ*_1_ and *τ*_2_, at which the lockdown is initiated and ended.

The epidemic dynamics are identical during those three periods except that the lockdown alters *R*_eff_(*t*, *τ*_1_, *τ*_2_) as follows.

Before the lockdown, $R_{0}^{1}=2.5$. During the lockdown, the reproductive rate if everyone were susceptible is $R_{0}^{2}=0.8$. After a sustained lockdown, that reproductive number would only bounce back to $R_{0}^{3}=2.0$, because some aspects of social distancing will be maintained indefinitely, or at least until a reliable vaccine is available, even though others will only be maintained during the lockdown. However, if the lockdown is short, then the post lock-down reproductive number would return to its original value of $R_{0}^{1}$. In particular, the gap between realized and potential value for $R_{0}^{3}$ decays exponentially in the length of the lockdown.

### COVID-19 deaths

For some people, COVID-19 is relatively mild, perhaps akin to a bad seasonal flu. We ignore costs associated with those cases, apart from recognizing that they cannot work while sick. Instead we focus on those who require hospitalization, particularly, those who require critical care.

If *p* denotes the proportion of infected people who need critical care, and *ξ*_1_ denotes the probability of death for those needing and receiving critical care, then *μ*_*I*_ = *pξ*_1_ *α*, where *α* is the reciprocal of the average duration of symptoms, which we take to be nine days.

That level of modeling precision is adequate for the state dynamics, because deaths from COVID-19 are not common enough to appreciably alter population size, but within the objective function greater precision is needed.

A central challenge of COVID-19 is the surges in demand for care, so we distinguish between those who need critical care and receive it from those who need it when hospitals are full and so cannot be treated properly. That means health harms are driven not only by the number of people who get infected, but also by how peaked the epidemic is; flattening the curve lets more people receive appropriate care.

In particular, we distinguish two components to the flow of deaths from COVID-19. In addition to deaths that are proportional to (*pI*(*t*)) there is an extra penalty term proportional to max({0, *pI* − *H*_max_}) where *H*_max_ is the number of critical care hospital beds available. The first term captures deaths that would occur even if there were no constraints on hospital capacity; the second captures the incremental risk of death if one needs critical care but does not receive it.

The max function is not differentiable. Furthermore, some costs arise when *pI* is smaller than but close to *H*_max_. For example, medical personnel (doctors, nurses) cannot give the usual care to individual patients if the intensive care unit is almost fully occupied. So we choose the following smooth function max(⋅, *ζ*) that is increasing and approximately linear in *pI*(*t*) − *H*_max_:

$$\begin{array}{c} \max\left(\left\{0,pI-H_{\max}\right\},\zeta \right)≔\frac{1}{\zeta }\log(1+e^{\zeta (pI\left(t\right)-H_{\max})}),\;\zeta \gg 1. \end{array}$$

**Fig 1:**
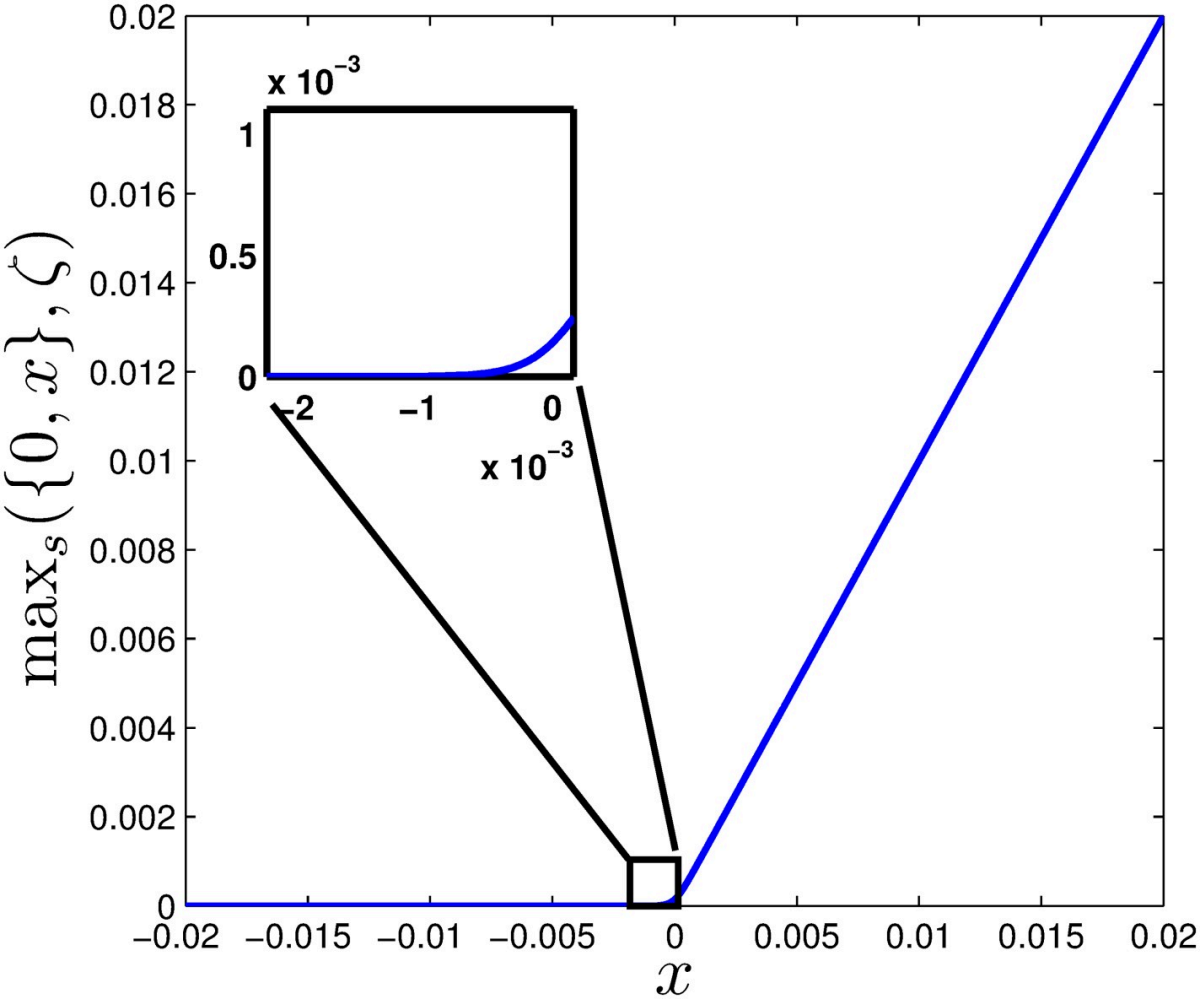
Approximation of the max function for *ζ* = 5000.

Fig 1 shows this is an extremely close approximation when *ζ* is large.

Hence, deaths from COVID-19 in the objective function can be written as

$$\begin{array}{c} \xi_{1}pI+\xi_{2}\max\left(\left\{0,pI-H_{\max}\right\},\zeta \right), \end{array}$$

where *ξ*_1_ is the death rate from COVID-19 of infected people who need and receive critical care, and *ξ*_2_ is the additional, incremental death rate when such individuals do not receive that care.

### Objective function

Deaths from COVID-19 are quick compared to those from chronic diseases or even cancer. The average time spent in hospital is about 12 days for those admitted to critical care and one-third that for others [14]. Since fatality rates are about 15% for all who are hospitalized and perhaps 45% for those receiving critical care, for any reasonable valuation of the social cost of a premature death, the cost of deaths is much greater than the cost of the hospitalization per se. Hence, health costs are represented by *M*, the cost per COVID-19 death, times the number of deaths.

There are instances of long-lasting morbidity associated with severe cases of COVID-19, with persistent damage to lungs or kidneys, for example. Should those turn out to be large compared to the costs of death, they could be accommodated through this same expression, just by using a larger value of *M*, if they are also driven by infections and unmet need for care.

Note this makes the health part of the objective function effectively piecewise linear in *I*. It is common to make cost be convex in an outcome, e.g., to use a quadratic function. However, that would imply the marginal cost of the 100th person who cannot receive needed care is greater than that of the 50th person. Making costs be (effectively) linear in the amount of unmet need values all people equally.

Economic activity is modeled as being proportional to the number of employed people raised to a power, as in a classic Cobb-Douglas model, with that exponent set to *σ* = 2/3 [15]. Since the model’s time horizon is so short, capital is presumed to be fixed and so is subsumed into the objective function coefficient *K* for economic activity.

Susceptible, latent, and recovered individuals are eligible to work (symptomatic individuals are assumed to be too sick to work, or are in quarantine), but because of the lockdown the actual number working is only a proportion *γ*(*t*) of those eligible to work *W*(*t*). Before the lockdown *γ*(*t*) = 1.0, during it is reduced to 0.25, and afterwards it bounces back but only partially. The longer the lockdown, the more jobs are lost semi-permanently because firms go out of business. That recovery is modeled as decaying exponentially in the length of the lockdown with a time constant of 0.001 per day, so that if a lockdown ended after six months, 17% of jobs suspended during the lockdown would not reappear, at least until a vaccine became available.

It is presumed that after a vaccine has been widely deployed, there will not again be many deaths from COVID-19, but the economy will not necessarily snap back to full employment instantly, so the objective function includes a salvage function to capture that. In particular, the salvage value function reflects the reduction in economic activity at time *T*, when a vaccine is deployed, relative to what it was at time 0 and so would have been but for COVID-19 and the lockdown. That is a rate or flow and needs to be multiplied by a factor Γ reflecting how long it takes for the economy to recover from that underemployment. No one knows the duration or shape of that recovery; which has variously been discussed as being V-shaped, U-shaped or W-shaped. For simplicity we set Γ = 365, as it would be for example if the recovery were linear but took two years, so the area is triangle-shaped.

#### Note

Omitting deaths after time *T* is a slight simplification, because some people who have an active infection at time *T* might still die after time *T*. However, because of the speed of the infection relative to the time horizon, it will be shown that the number of people infected at time *T* is not so large, so this is not a major concern.

### Full model

The decision variables are *τ*_1_ and *τ*_2_, the times when the lockdown begins and ends, and the full model can be written as:

$$\begin{array}{ll} V(X_{0},\tau_{1},\tau_{2})≔ & \left(2\text{a}\right)\\ \int_{0}^{T}(M(\xi_{1}pI(t)+\xi_{2}\text{max}(\{0,pI(t)-H_{\text{max}}\},\zeta ))-K\gamma {\left(t,\tau_{1},\tau_{2}\right)}^{\sigma }W{\left(t\right)}^{\sigma })dt+ & \left(2\text{b}\right) \end{array}$$

$$\begin{array}{cc} & \left(T+\Gamma \right)KW{\left(0\right)}^{\sigma }\gamma {\left(0,\tau_{1},\tau_{2}\right)}^{\sigma }-\Gamma KW{\left(T\right)}^{\sigma }\gamma {\left(T,\tau_{1},\tau_{2}\right)}^{\sigma } \end{array}$$ (2c)

$$\begin{array}{cc} & V^{*}\left(X_{0}\right)≔\min_{\tau_{1},\tau_{2}}V\left(X_{0},\tau_{1},\tau_{2}\right),\;X≔\left(S,L,I,R\right),\;W≔S+L+R. \end{array}$$ (2d)

$$\begin{array}{cc} \text{s.t.}\; & \dot{X}\left(t\right)=\{\begin{array}{cc} {\mathrm{SLIR}}_{1}\left(X\left(t\right),\tau_{1},\tau_{2}\right) & 0\leq t<\tau_{1}\\ {\mathrm{SLIR}}_{2}\left(X\left(t\right),\tau_{1},\tau_{2}\right), & \tau_{1}\leq t\leq \tau_{2}\\ {\mathrm{SLIR}}_{3}\left(X\left(t\right),\tau_{1},\tau_{2}\right) & \tau_{2}<t\leq T \end{array} \end{array}$$ (2e)

$$\begin{array}{cc} & X\left(0\right)=X_{0}\geq 0 \end{array}$$ (2f)

$$\begin{array}{cc} & \gamma \left(t,\tau_{1},\tau_{2}\right)≔\{\begin{array}{cc} \gamma_{1} & 0\leq t<\tau_{1}\\ \gamma_{2} & \tau_{1}\leq t\leq \tau_{2}\\ \gamma_{3}\left(\tau_{1},\tau_{2}\right)≔\gamma_{2}+\left(\gamma_{1}-\gamma_{2}\right)e^{\kappa_{2}\left(\tau_{1}-\tau_{2}\right)} & \tau_{2}<t\leq T \end{array} \end{array}$$ (2g)

$$\begin{array}{cc} & R_{\text{eff}}\left(t,\tau_{1},\tau_{2}\right)≔\{\begin{array}{cc} R_{0}^{1} & 0\leq t<\tau_{1}\\ R_{0}^{2} & \tau_{1}\leq t\leq \tau_{2}\\ R_{0}^{3}\left(\tau_{1},\tau_{2}\right)≔{\bar{R}}_{0}^{3}+\left(R_{0}^{1}-{\bar{R}}_{0}^{3}\right)e^{\kappa_{1}\left(\tau_{1}-\tau_{2}\right)} & \tau_{2}<t\leq T \end{array} \end{array}$$ (2h)

$$\begin{array}{cc} & \text{with}\;R_{0}^{2}\leq {\bar{R}}_{0}^{3}\leq R_{0}^{1}. \end{array}$$ (2i)

To refer to the health care term, the economic (labor) term, and the salvage functions the objective value (2c) we shortly write

$$\begin{array}{c} V_{h}\left(X_{0},\tau_{1},\tau_{2},M\right)≔M\int_{0}^{T}(\xi_{1}pI+\xi_{2}\max\left(\left\{0,pI-H_{\max}\right\},\zeta \right))dt \end{array}$$ (2j)

$$\begin{array}{cc} & V_{l}\left(X_{0},\tau_{1},\tau_{2},K\right)≔TKW{\left(0\right)}^{\sigma }\gamma {\left(0,\tau_{1},\tau_{2}\right)}^{\sigma }-K\int_{0}^{T}\gamma {\left(t,\tau_{1},\tau_{2}\right)}^{\sigma }W{\left(t\right)}^{\sigma }dt \end{array}$$ (2k)

$$\begin{array}{cc} & V_{s}\left(T,\tau_{1},\tau_{2},K\right)≔\Gamma KW{\left(0\right)}^{\sigma }\gamma {\left(0,\tau_{1},\tau_{2}\right)}^{\sigma }-\Gamma KW{\left(T\right)}^{\sigma }\gamma \left(T,\tau_{1},\tau_{2}\right). \end{array}$$ (2l)

The derivation of the necessary optimality conditions can be found in the Appendix 1. The Matlab toolbox OCMAT is used for the numerical calculations. See http://orcos.tuwien.ac.at/research/ocmat_software.

### Parameterization

The initial population is normalized to 1.0. Optimization begins with *L*(0) = 0.1% of the population being latent and 99.9% being susceptible and continues over a finite time horizon of *T* = 365 days, representing the time until a vaccine is hoped to be widely available.

The qualitative results are similar with *T* = 730 days.

Table 1 summarizes the base case parameters, several of which have already been discussed. The others are addressed here.

**Table 1:** Base case parameter values.

*α*	$R_{0}^{1,2.3}$	*H*_max_	*p*	*M*	*K*	*γ*_1,2,3_	Γ	*φ*	*f*
$\frac{1}{9}$	2.5, 0.8, 2.0	1.76 × 10^−4^	2.311 × 10^−2^	21716	1	1, 0.25, 0.75	365	$\frac{1}{7.2}$	0.75
*ω*	*κ*_1_	*κ*_2_	*σ*	*μ*	*ν*	*μ*_*I*_	*ζ*	*ξ*_1_	*ξ*_2_
0.6	2 × 10^−3^	10^−3^	$\frac{2}{3}$	$\frac{0.01}{365}$	$\frac{0.01}{365}$	$\frac{13}{10800}$	5000	0.05	$\frac{0.55}{9}$

In July 2020, the U.S. Centers for Disease Control (2020) released new guidance for parameters in COVID-19 planning models. They suggest assuming that *ω* = 60% of infections become symptomatic, that infectiousness in the *L* state is *f* = 75% of that in the *I* state. They suggest that presymptomatic individuals spend 6 days in the *L* state, so we make *α* = 1/9, corresponding to a dwell time of 9 days in the *I* state, so symptomatic cases are infectious for a total of 6 + 9 = 15 days.

The CDC advises that 50% of infections arise from people not manifesting symptoms (i.e., from the *L* state), and that implies that those who are never symptomatic remain infectious for 9 days. (Since that makes the average number of infections per person passing through the *L* state, or (*ω* ⋅ 6 + (1 − *ω*) ⋅ 9) × 75% match the corresponding figure for infections coming from the *I* state.) That in turn implies that the average dwell time in the *L* state is *ω* ⋅ 6 + (1 − *ω*) ⋅ 9 = 7.2 days, so *φ* = 1/7.2.

The CDC estimates of the probabilities of death given hospitalization vary by age, but the weighted average, weighting by the age distribution of hospital cases, is roughly 15%. Dividing that by their estimate that 32% of COVID-19 hospital admissions need intensive care (ICU) suggests that the probability of death for those needing and receiving intensive care is about 45%. The parameter *ξ*_1_ is the death rate per day for such people. Since the average dwell time in the *I* state is 9 days, the death rate per day is *ξ*_1_ = 5%.

*ξ*_2_ is the additional, incremental death rate per day for infected people who need critical care but do not receive it. If the death rate for such individuals over an entire infection is 100% and the average dwell time in the *I* state is 9 days, then the incremental death rate per day is *ξ*_2_ = *α*(1 − 45%), or about 6.11%.

ICU beds are a smaller share of all hospital beds than is the proportion of COVID-19 hospital admissions needing ICU care, even after adjusting for different lengths of stay, so the care constraint will be on critical care beds.

[16] suggest that in the U.S., 58,166 of the existing 84,750 ICU beds could be made available for treating COVID-19 patients. Given the U.S. population is about 330 million, that is 0.176 per 1,000 people. The model acts as if patients who need critical care at some point need that care throughout their 9-day dwell time in the *I* state. CDC data suggest that the average time in hospital for those needing critical care at some point is about 12 days, but not all of that time is necessarily in the ICU, so the 9 days is probably about right. So we set *H*_max_ = 176 per 1,000 or 0.000176. There are roughly ten times as many hospital beds as critical care beds, so sensitivity analysis with larger values of *H*_max_ may be of interest if some regular beds could be converted over to critical care beds, although some readers have been skeptical about that possibility.

The probability of needing ICU care for those entering the *I* state, *p*, is the proportion of hospital admissions needing ICU care (32%) times the proportion of those showing symptoms who need hospitalization. The latter quantity can be computed indirectly because the infection fatality rate (which CDC gives as 0.65%) is the product of that quantity, the probability of death given hospitalization (15%) and the proportion of cases that are symptomatic (*ω* = 60%). That implies that the probability of hospitalization given symptoms is 0.65%/(15% ⋅ 60%) = 7.22%, and so *p* = 7.22% ⋅ 32% = 2.31%.

Dividing the infection fatality rate (0.65%) by *ω* = 60% implies that the probability of death given symptoms is 1.0833%. Dividing that by the average dwell time in the *I* state implies that *μ*_*I*_ = 0.0012037.

There is not truly consensus in the literature about any of the key parameters, but the two for which the widest range of values seem plausible are the proportion of symptoms that become symptomatic, *ω*, and the social cost of a death, *M*, so we discuss them at length.

The CDC’s estimate that 60% of infections produce enough symptoms to be detected reflects the original understanding of the epidemic, but there have recently though been community-wide antibody tests in several European countries, in New York State, Santa Clara and Los Angeles Counties in California, and in a number of prisons, all suggesting that there may be far more undetected infections than was previously thought. For example, the Santa Clara study concluded that the actual number infected could be 50 to 85 times more than the number of confirmed cases [17].

That would suggest a substantially lower level of *ω* may be appropriate. [18], considering data from multiple European countries, suggest that infections could be ten times more common than previously supposed. That may be overstating, but we consider values down to *ω* = 12% in other runs of the model, and the results below include sensitivity analysis with respect to this parameter.

Estimating the relative value of lost work vs. lost lives is tricky, to say the least, so without loss of generality we set *K* = 1 and consider a very wide range of values for *M*.

Still, it is helpful to determine at least roughly the size of *M* relative to *K*. [7] value a premature death at 20 × GDP per capita, while noting that [19] use a much greater value of 150 × GDP per capita. [20] offers multiple reasons why lower values may be preferred for analysis of COVID-19 in particular. For example, lower values would apply if one focused on years-of-life-lost, since most deaths are among the elderly, especially those with other pre-existing conditions. E.g., [21] report that the vast majority of those hospitalized for COVID-19 had prior serious comorbidities such as hypertension, obesity, and diabetes, to the extent that their estimated 10-year survival rate absent COVID-19 was only 53%. So we consider a range from 10 × to 150 × GDP per capita.

*K*(*γW*)^2/3^ measures GDP per day—*K* is the constant that we assume to capture everything except labor, so 365 *K*(*γW*)^2/3^ equals the nation’s GDP. Since the population size is normalized to 1.0, that implies values of a premature death, *M*, somewhere in the range from 3,650 to 54,750. We set *M* = 21, 716 for analyses with a fixed *M*.

## Results

### Optimizing only over the end time, *τ*_2_

**Fig 2:**
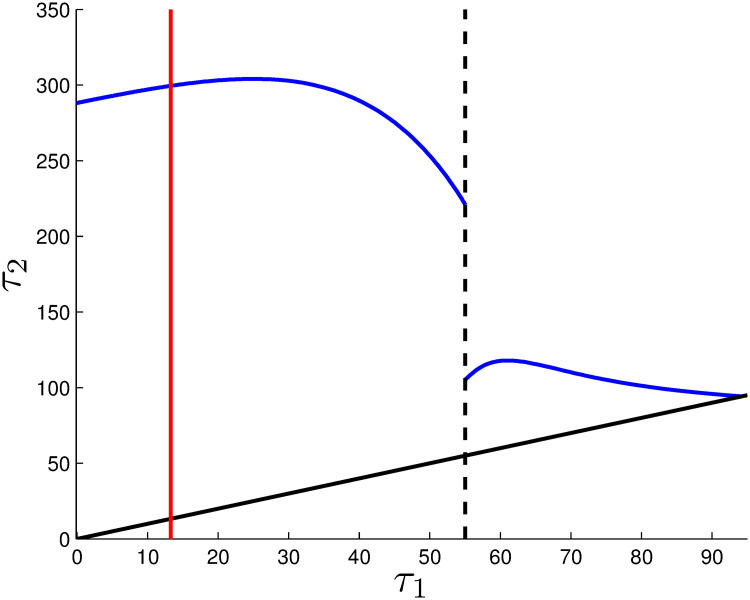
Fixed initial lockdown time *τ*_1_ and optimally chosen time *τ*_2_. For *τ*_1_ = 44 there exists a Skiba solution, i.e. there are two different solution paths which deliver the same objective value.

Countries have already started their lockdowns, so we start by optimizing only over *τ*_2_, but for various values of *τ*_1_ because different places started their lockdowns at different times. Fig 2 plots the resulting optimized value of *τ*_2_ (blue curve) vs. *τ*_1_. For convenience, the value of *τ*_1_ is also shown, by the black line, so it is easy to visualize the duration of the lockdown as the height of the gap between the blue and black lines.

As *τ*_1_ increases from 0 to a little more than 25, *τ*_2_ increases by about the same amount, keeping the duration of the lockdown roughly constant. That makes intuitive sense. The later one begins the lockdown, the later it should be relaxed.

However, as *τ*_1_ increases further, from about 25 to 44, the optimal *τ*_2_ decreases. Hence, over that range, countries that started their lockdown later should end it sooner, something that is perhaps surprising. It may be that the timing of the ending of the lockdown is related to the accumulation of infections, which happens rapidly when the lockdown is not initiated early.

The solid red vertical line indicates the value of *τ*_1_ that produces the lowest total cost, when *τ*_2_ is optimized, and that is for *τ*_1_ = 3.92.

That this optimal value of *τ*_1_ is greater than zero is perhaps a second surprising result. If lockdowns are costly, in this case because they reduce work, then it is possible to start a lockdown too soon, and for a later starting date to be preferred. Since the epidemic grows explosively, even exponentially, before the lockdown, some might have thought that earlier was always better.

Perhaps not surprisingly, if the initial reproductive rate $R_{0}^{1}$ is smaller then this optimal starting time comes later. This may explain some of the tension observed between residents of rural areas and urban areas over lockdown timing. The optimal date for starting the lockdown in a place like New York City, with high population density (so likely higher $R_{0}^{1}$) and early onset could be sooner than the optimal date for starting the lockdown elsewhere in the country.

When *τ*_1_ equals the critical value of 44, indicated by the vertical dashed line, there are two alternative optimal solutions for *τ*_2_. Both *τ*_2_ = 222.5 (lockdown for over 7 months) and *τ*_2_ = 86.9 (a lockdown of less than three months) produce the same objective function value. And as *τ*_1_ increases beyond 44, the best end time for the lockdown decreases further. Eventually, if the lockdown doesn’t start until day 84, it is optimal not to start a lockdown at all.

The interpretation for this is that there are two broad strategies that can be pursued. One, might be called an ‘eradication’ strategy. It locks down long enough to push infections down to minimal levels, with just a modest rebound shortly before the end of the time horizon when a vaccine becomes available. The second, which might be called ‘curve flattening’, uses the lockdown to reduce the size of the initial spike in infections, so fewer come when infections exceed hospital capacity. The later the lockdown starts, the harder it is to pull off the eradication strategy, until at some critical point (in this case *τ* = 44) it becomes optimal to switch to the ‘curve flattening’ strategy which requires a much shorter lockdown.

**Fig 3:**
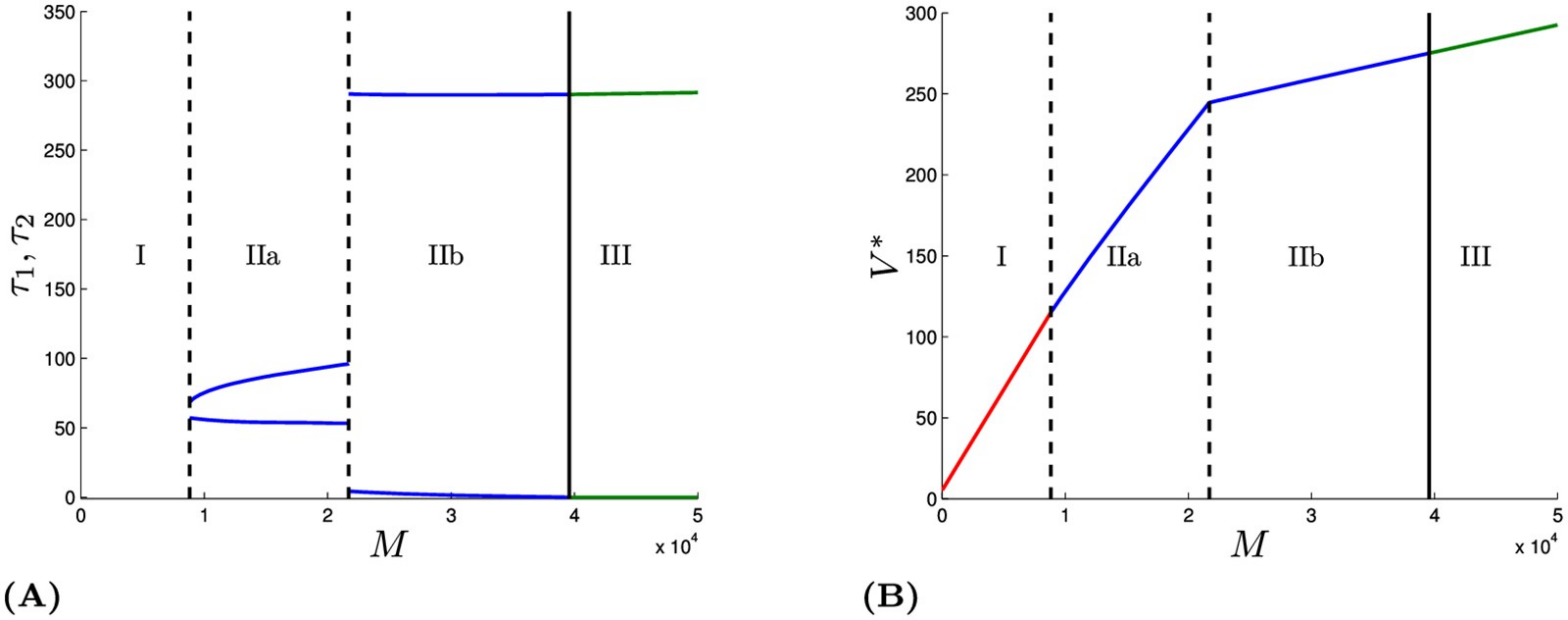
Figure showing the different regimes for varying *M*. The other parameter values are taken from Table 1. Mathematical description of the three regimes: Regime I: no lockdown, i.e. $\dot{X}\left(t\right)={\mathrm{SLIR}}_{1}\left(t\right),0\leq t\leq 365$, Regime II: lockdown in interval (0, 365), i.e. 0 < *τ*_1_ < *τ*_2_ < 365, Regime III, lockdown starts immediately, i.e. 0 = *τ*_1_ < *τ*_2_ < 365.

Optimizing over both *τ*_1_ and *τ*_2_ makes these results easier to understand. Fig 3 shows the optimal values of *τ*_1_ and *τ*_2_ (left panel) and the optimal solution value V* (right panel) as a function of *M*, the cost of a COVID-19 death. It shows three regions. In region I, on the far left when *M* is very small, it is optimal to never lockdown and just let the epidemic run its course. Basically, if one does not care much about deaths, beyond their effect on economic productivity from reducing work, then the lockdown is not worth it.

In Region III, corresponding to large values of *M*, one should begin the lockdown immediately and keep it in place until shortly before the vaccine becomes available.

In Region II both broad strategies (flattening and eradication) can be considered, with flattening being preferred in Region IIa, eradication being preferred in Region IIb, and either being optimal when *M* is just exactly at the value separating Regions IIa and IIb. At that *M* = 21, 716, there is a so-called Skiba point.

The existence of the Skiba point implies another surprising result. Both short and long lockdowns can be optimal for the exact same set of parameter values. Exact equality only occurs at that specific value of *M*, but for a range of values in that neighborhood, the two very different strategies perform nearly as well. So a single person can be indifferent, or nearly indifferent, between two very different approaches.

Furthermore, two people with modestly different relative valuations on work and health can favor very different policies, if their modestly different values of *M* lie on either side of the Skiba threshold.

As mentioned, plausible values of *M* range from 3,650 to 54,750. The lower end of that range lies within Region I, but increasing *M* within that range would carry the solution through Regions IIa and IIb, all the way into Region III. As mentioned earlier, we focus on qualitative results, not specific values. So we do not think the model shows that one strategy or another is necessarily the best. Rather, we would say that the model suggests that it is hard to be certain about that. Any one of the strategies could plausibly turn out to be optimal, depending on how uncertainty about the parameter values is resolved.

**Fig 4:**
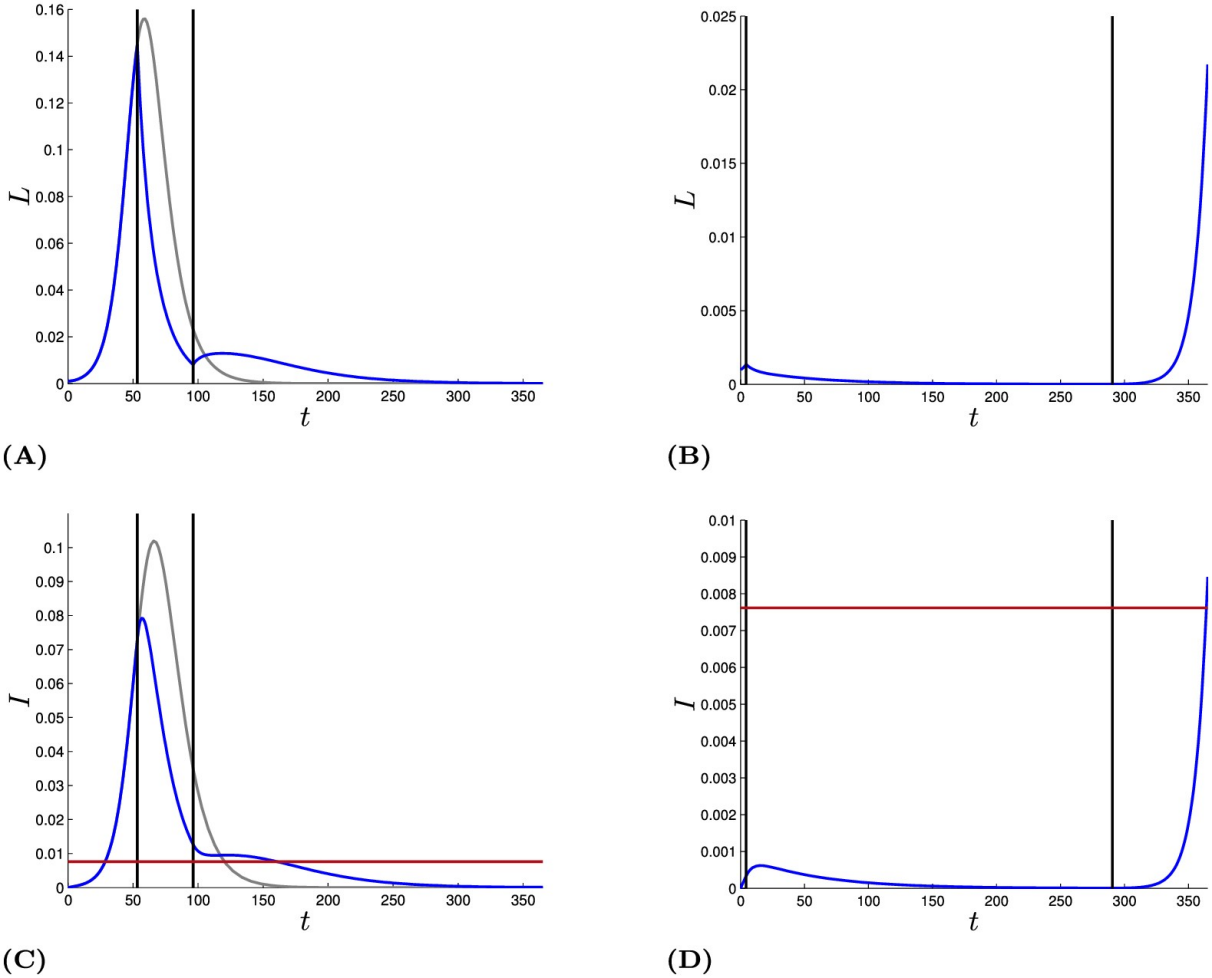
Panel (A) and (B) show the two different solutions paths emerging from the Skiba point for the number of latent *L*(⋅) (blue lines) and panel (C) and (D) show those paths for the number of infected *I*(⋅). The gray line in panels (A) and (C) depicts the uncontrolled path without lockdown for comparison. For the total costs and its shares see Table 2. The horizontal red line shows the health care capacity *H*_max_/*p*.

Fig 4 contrasts the two solutions when *M* is exactly at its Skiba threshold value of 21, 716. The vertical lines indicate the start (*τ*_1_) and end (*τ*_2_) times of the lockdown for the curve flattening strategy (dashed lines; left panel) and the eradication strategy (dashed lines; right panel). The blue lines show the numbers who are infected under those two strategies, while the gray line shows the uncontrolled epidemic with no lockdown.

The number in the latent state tracks closely the number in the infected state. In particular, the number who are infected and symptomatic is a slightly lagged and smoothed parallel to the number in the latent state. The number of infected is lower than the number in the latent state in part because of the higher death rate for those who are infected, but primarily because the latent state includes asymptomatic as well as presymptomatic individuals.

Note the very different vertical scales in the two panels. The eradication strategy keeps the number of infections well below the point where hospital capacity becomes binding, whereas the curve flattening strategy (greatly) exceeds that capacity, at times.

Here the optimal curve flattening strategy experiences a second, much smaller bump, but for other parameter values it can be a second spike. That is, it can be optimal to end the lockdown in a way that creates a resurgence of the epidemic. That the epidemic spikes again after ending a lockdown does not imply that ending the lockdown was a mistake.

Table 2 contrasts the valuations of the three components of the objective function under the two strategies, and the uncontrolled epidemic as a foil. The values can perhaps best be understood in percentage terms. In the absence of an epidemic, economic output would have been 365, and in the absence of controls the health cost would be 270. So under the shorter lockdown, economic output falls by about 9% for the year, and the costs of premature deaths are reduced by about one-quarter. With the longer lockdown, 86% of the health costs are averted, but economic output falls by nearly 50%.

**Table 2:** The total costs, the share of costs of the health care system, profit from labor and the salvage value for the Skiba solution and the uncontrolled solution of Fig 4 (*M* = 21, 716, *K* = 1) and the lockdown times.

	Flattening	Eradication	Uncontrolled
V*(*X*_0_)	244.6	244.6	275.2
*V*_*h*_(*X*_0_, *τ*_1_, *τ*_2_, *M*)	206.8	38.0	269.9
*V*_*l*_(*X*_0_, *τ*_1_, *τ*_2_, 1)	31.9	177.9	4.1
*V*_*s*_(*T*, *τ*_1_, *τ*_2_, *K*)	5.9	28.7	1.2
[*τ*_1_, *τ*_2_]	[53.3, 96.2]	[4.4, 290.5]	

### Sensitivity analysis with respect to *M*

**Fig 5:**
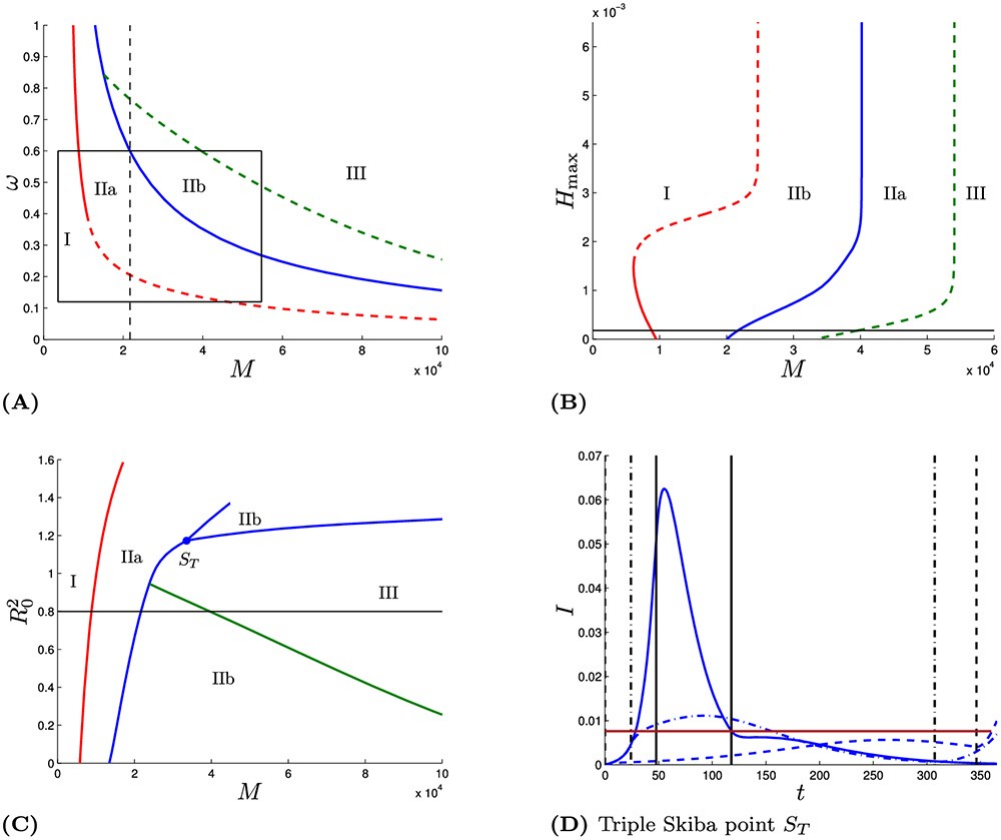
Bifurcation diagrams. Dashed curves correspond to a continuous regime transition and solid lines to a disruptive regime transition. Panel (A) shows the bifurcation diagram for parameter values of Table 1 while varying *ω* and *M*. Panel (B) shows the bifurcation diagram for parameter values of Table 1 and varying *H*_max_ and *M*. Panel (C) shows the bifurcation diagram for *M* and $R_{0}^{2}$. The black horizontal lines depict base case parameter valued. The solid vertical lines in panel (B) delimit the region *M* ∈ [0.365 × 10^3^, 5.475 × 10^4^]. The dashed vertical line shows the *M* value of the base case (Skiba case). In panel (D) a triple Skiba solution is depicted (resulting for the $\left(M,R_{0}^{2}\right)$-parameter combination *S*_*T*_ of panel (C)). In that case two “eradication” solutions and one “curve flattening” solutions exist. The dashed solution starts with quarantine immediately.

Fig 5 is a sensitivity analysis with respect to the value of *M*, i.e., the relative valuation placed on health as opposed to economic outcomes.

The upper left panel shows which type of solution is optimal for various values of the two most uncertain parameters, the proportion of infections that become symptomatic (*ω*) and the value of averting a death, expressed as a multiple of the nation’s daily GDP per capita (*M*). Even holding *ω* at its base case value of 60% and varying *M* between 0.365 and 5.475 × 10^4^ spans all four regions. Adding in the variation in *ω* down to its lower plausible value of 12% just accentuates the uncertainty as to which region pertains. That indicates that people can disagree about the best broad strategy depending on their judgments about how to balance health and economic outcomes and on the degree to which the new antibody testing results alter judgments about how many infections remain undetected. This shows that two reasonable, intelligent people who agree completely on how to think about the epidemiology, health, and economics of the COVID-19 crisis can still reach very different conclusions about what policy is best, just because of differences concerning these two key parameters, about which there is no scientific agreement, at least at present.

That upper left panel is drawn for *H*_max_ = 0.000176. The upper right panel of Fig 5 is a bifurcation diagram showing which strategies are optimal as a function of both *M* and *H*_max_, which represents the number of critical care beds, while holding *p* at the value of 0.0231. That diagram shows that the qualitative results would not change until *H*_max_ increased to a very large multiple of its base case value of 0.000176. For the most part, the curves in that panel slope up and to the right, meaning that if treatment capacity and the valuation of a life both increase, the structure of the solution remains the same. Eventually those curves turn vertical when hospital capacity is no longer binding.

The exception is that for *M* small we have, for increasing *H*_max_, first Region I then IIa and then I again. This curious pattern can be explained as follows. Because *M* is small, lockdowns will be short, if they exist at all, and so most people will become infected. If *H*_max_ is very small then almost all of those infections will occur when hospital capacity has been exceeded. Conversely, if *H*_max_ is very large, then almost all infections will occur when there is adequate hospital capacity, even if there is no lockdown. It is only for intermediate values of *H*_max_ that the lockdown has the ability to tip a meaningful share of the infections from times when hospital capacity is exceeded to times when adequate care can be provided.

The lower left panel shows how the optimal solutions depend on both $R_{0}^{2}$ and *M* when *p* is fixed at the value 0.0231. Not surprisingly, lockdowns are preferred when *M* is large and/or $R_{0}^{2}$ is low, which explains why the boundaries are generally upward sloping.

Within the range of $R_{0}^{2}$ of greatest interest, the boundaries between no lockdown and short lockdown, and between short and long lockdowns are almost vertical, indicating that the precise value of $R_{0}^{2}$ is less important than the value of *M* for driving what strategy is optimal.

An exception is the curve between regions IIb and III. That is because for very low values of $R_{0}^{2}$, the lockdown is so effective that it is not necessary to start the lockdown immediately to fight the epidemic.

For $R_{0}^{2}=1.1724$ and *M* = 33, 550 there is a so-called triple Skiba point, where three qualitatively different strategies perform equally well. The lower right panel depicts the time trajectories for *I*(*t*) under those three strategies which are: (1) Locking down immediately (dashed line), (2) Locking down around day 24 and holding that nearly until the vaccine arrives (dot-dash lines), and (3) merely flattening the curve with a relatively short lockdown from *τ*_1_ = 48 to *τ*_2_ = 118. Infections increase initially after lockdown under the two eradication strategies because $R_{0}^{2}$ is greater than one at that triple Skiba point.

One additional sensitivity analysis (not shown) considered the scenario when treatment is much more effective, so that the probability of dying if one needs and receives critical care is only 4.5% not 45%. That makes the extra penalty for exceeding hospital capacity gets much worse, 95.5% not 55%, but this does not change much about the structure of the solution.

## Conclusions

### Primary findings

This paper is about planning the timing of complete lockdowns in COVID-19 times. The aim of installing a lockdown is to limit social contact to reduce the number of people getting infected. The drawback is the concurrent reduction in economic activity. The latter also has an effect after the lockdown has ended, because it takes time for the economy to recover.

Our model is based on the epidemiological SLIR dynamics, which we combine with a basic Cobb-Douglas model of economic activity to develop a framework that can evaluate the above-described tradeoff. In many countries the number of COVID-19 patients needing intensive care treatment came close to or even exceeded the available intensive care capacity, so that aspect is included in our model.

We find that essentially two different solution patterns can be optimal. One is what we call an *eradication strategy*, where a long lockdown significantly reduces not only the bad health effects of the epidemic but also economic activity. The other is a *curve flattening strategy* characterized by a relatively short lockdown period. The idea is to reduce the peak of the number of infected in order to limit the violation of the intensive care capacity constraint, where at the same time economic activity is not harmed as much.

Interesting is that the performance of both strategies can be quite similar. Indeed, there are specific parameter values such that two decision makers with the same preferences for health versus the economy can opt for completely different lockdown policies and both be choosing optimally.

We note that over the plausible ranges for two key parameters, namely the proportion of infections that become symptomatic and the valuation placed on preventing a COVID-19 related death, all types of solutions can be optimal. We view that as indicating that a degree of humility and open-mindedness may be appropriate; at least in our model, it is not necessarily clear whether a longer or a shorter lockdown is best.

### Limitations

This model simplifies in many respects that could bear on the optimal timing and duration of a lockdown. That is why we stress qualitative results, not numerical results. These limitations include:

- Transmission mitigation is modeled crudely as a lockdown being in place or not. The intensity of the lockdown could instead be modeled as a continuous control variable because there are a wide range of non-pharmaceutical interventions that could be deployed in various combinations, not just all-or-nothing (see [22] and [23]). That would permit easing the lockdown gradually. In the present model the lockdown ends abruptly, although not completely; we model the reproductive rate as bouncing back to 2.0 but not its pre-lockdown level of 2.5 because we presume some protective measures are retained even after people go back to work. Also, the most important innovations might be efforts to re-engineer operations to make them safer, rather than either shutting them down or allowing them to operate normally.
- This model does not divide the population by gender, age or pre-existing health condition. Death rates are sharply higher for those who are older or who have pre-existing conditions. Strategies that have longer or more restrictive lockdowns for vulnerable populations may be prudent but cannot be modeled here. So the findings are directly relevant when policy is constrained not to impose different lock downs on different socioeconomic groups.
- The technology for treating COVID-19 cases may improve over time. E.g., Remdesivir and convalescent plasma treatments are now being used. Improving technology could favor earlier and longer lockdowns if that defers cases until technology is better.
- The model does not consider seasonality. That may or may not be a serious limitation. The virus clearly rebounded sharply in many places during the Northern Hemisphere’s fall, but our understanding is that there is still some debate as to whether that is seasonality per se, or the lagged effect of relaxation of policies and ensuing vacationing. Still, there are variations over time in rates of social interaction, e.g., because of holidays and school calendars, and those are not considered at present.
- The model does not consider the possibility of long-term health effects of survivors of COVID-19, although there are reports of lasting harm not only to lungs, but also to the brain and kidney.
- The model ignores geography. In reality there is not one social planner governing one region, but multiple regions that affect each others’ economies and epidemics. The optimal policy for one region may depend on what is happening in other regions to which it is connected by travel and migration, in terms of infection rates, policy choices, and economic dislocation.

### Further work

A variety of extensions of this model are possible, including making the timing of the vaccine’s arrival unknown, allowing for multiple lockdowns separated by periods of relaxation, and altering the epidemic’s dynamics when the number of new infections is small enough that contact tracing can lead to quickly quarantining everyone that an incident case might have infected (see [24] and [25]).

The epidemic model could be enriched in various ways including making the rates of social interaction a distributed parameter that varies across a heterogeneous population, and modeling explicitly the population’s patience with and commitment to lockdown restrictions.

### Appendix 1

#### Necessary optimality conditions

Setting *τ*_0_ ≔ 0 and *τ*_3_ ≔ *T* the Hamiltonians for the three stages *i* = 1, 2, 3 are

$$\begin{array}{c} H_{i}\left(X_{i},\Lambda_{i},\tau_{1},\tau_{2}\right)≔M(\xi_{1}pI+\xi_{2}\max\left(\left\{0,pI-H_{\max}\right\},\zeta \right)-K\gamma {\left(t,\tau_{1},\tau_{2}\right)}^{\sigma }W{\left(t\right)}^{\sigma })+\\ \Lambda_{i}^{{}^{\prime }}{\mathrm{SLIR}}_{i}\left(X_{i},\tau_{1},\tau_{2}\right), \end{array}$$ (3)

with

$$\begin{array}{cc} & X_{i}\left(t\right)≔X\left(t\right),\;\tau_{i-1}\leq t\leq \tau_{i}\\ & \Lambda_{i}\left(t\right)≔\left(\lambda_{1}\left(t\right),\lambda_{2}\left(t\right),\lambda_{3}\left(t\right),\lambda_{4}\left(t\right)\right),\;\tau_{i-1}\leq t\leq \tau_{i}. \end{array}$$

The costates satisfy the canonical system

$$\begin{array}{c} {\dot{\Lambda }}_{i}\left(t\right)=-\frac{\partial }{\partial X_{i}}H_{i}\left(X_{i}\left(t\right),\Lambda_{i}\left(t\right),\tau_{1},\tau_{2}\right),\;\tau_{i-1}<t<\tau_{i}. \end{array}$$ (4)

Since the RHS of the ODEs (4) are continuously differentiable, the costates can continuously be extended to the left and right side of the interval *τ*_*i*−1_ < *t* < *τ*_*i*_. Thus

$$\begin{array}{c} \Lambda_{i}\left(\tau_{i-1}\right)≔\lim_{t\to \tau_{i-1}^{+}}\Lambda_{i}\left(t\right)\;\text{and}\;\Lambda_{i}\left(\tau_{i}\right)≔\lim_{t\to \tau_{i}^{-}}\Lambda_{i}\left(t\right) \end{array}$$

uniquely exist. For the derivatives of the Hamiltonians with respect to the switching times we find

$$\begin{array}{cc} & \frac{\partial }{\partial \tau_{1}}H_{i}\left(X_{i},\Lambda_{i},\tau_{1},\tau_{2}\right)=\{\begin{array}{cc} 0 & i=1,2\\ \gamma_{3}\left(\tau_{1},\tau_{2}\right)K\sigma \gamma_{3}{\left(\tau_{1},\tau_{2}\right)}^{\sigma -1}W^{\sigma }+\Lambda_{i}^{{}^{\prime }}\frac{\partial }{\partial \tau_{1}}{\mathrm{SLIR}}_{i}\left(X_{i},\tau_{1},\tau_{2}\right) & i=3 \end{array}\\ & \frac{\partial }{\partial \tau_{2}}H_{i}\left(X_{i},\Lambda_{i},\tau_{1},\tau_{2}\right)=\{\begin{array}{cc} 0 & i=1,2\\ -\gamma_{3}\left(\tau_{1},\tau_{2}\right)K\sigma \gamma_{3}{\left(\tau_{1},\tau_{2}\right)}^{\sigma -1}W^{\sigma }+\Lambda_{i}^{{}^{\prime }}\frac{\partial }{\partial \tau_{2}}{\mathrm{SLIR}}_{i}\left(X_{i},\tau_{1},\tau_{2}\right) & i=3 \end{array} \end{array}$$

The Hamiltonian in the third stage (after the lockdown) and the salvage value explicitly depend on the switching times *τ*_1_ and *τ*_2_. Thus for 0 < *τ*_1_ < *τ*_2_ < *T* the necessary optimality conditions at the switching times *τ*_1_ and *τ*_2_ write as (cf. [26]).

$$\begin{array}{ccc} H_{2}\left(X_{2}\left(\tau_{1}\right),\Lambda_{2}\left(\tau_{1}\right),\tau_{1},\tau_{2}\right)-H_{1}\left(X_{1}\left(\tau_{1}\right),\Lambda_{1}\left(\tau_{1}\right),\tau_{1},\tau_{2}\right) & = & \int_{\tau_{2}}^{T}\frac{d}{d\tau_{1}}H_{3}\left(X_{3}\left(t\right),\Lambda_{3}\left(t\right),\tau_{1},\tau_{2}\right)dt+\\ & & K\frac{d}{d\tau_{1}}\gamma \left(T,\tau_{1},\tau_{2}\right)\\ H_{3}\left(X_{3}\left(\tau_{2}\right),\Lambda_{3}\left(\tau_{2}\right),\tau_{1},\tau_{2}\right)-H_{2}\left(X_{2}\left(\tau_{2}\right),\Lambda_{2}\left(\tau_{2}\right),\tau_{1},\tau_{2}\right) & = & \int_{\tau_{2}}^{T}\frac{d}{d\tau_{2}}H_{3}\left(X_{3}\left(t\right),\Lambda_{3}\left(t\right),\tau_{1},\tau_{2}\right)dt+\\ & & K\frac{d}{d\tau_{2}}\gamma \left(T,\tau_{1},\tau_{2}\right) \end{array}$$

and at *τ*_*i*_, *i* = 1, 2 the costates satisfy

$$\begin{array}{c} \Lambda_{1}\left(\tau_{1}\right)=\Lambda_{2}\left(\tau_{1}\right)\\ \Lambda_{2}\left(\tau_{2}\right)=\Lambda_{3}\left(\tau_{2}\right). \end{array}$$

At the endtime *T* the costates satisfy the transversality condition

$$\begin{array}{c} \Lambda \left(T\right)=(\begin{array}{c} \lambda_{1}\left(T\right)\\ \lambda_{2}\left(T\right)\\ \lambda_{3}\left(T\right)\\ \lambda_{4}\left(T\right) \end{array})=(\begin{array}{c} -\Gamma K\sigma W{\left(T\right)}^{\sigma -1}\gamma {\left(T,\tau_{1},\tau_{2}\right)}^{\sigma }\\ -\Gamma K\sigma W{\left(T\right)}^{\sigma -1}\gamma {\left(T,\tau_{1},\tau_{2}\right)}^{\sigma }\\ 0\\ -\Gamma K\sigma W{\left(T\right)}^{\sigma -1}\gamma {\left(T,\tau_{1},\tau_{2}\right)}^{\sigma } \end{array}). \end{array}$$

### Appendix 2

#### Derivation of *R*_0_

For the derivation of the reproductive rate *R*_0_ of the SLIR model (1) we assume *ν* = *μ*. Then, for every *S* there exists a disease free equilibrium (*S*, 0, 0, 0) of system (1). Using the expansion formula for the determinant along the first column it can easily be seen that the stability of this equilibrium only depends on the dynamics of the latent and infected population. Thus, we consider the linearization of the *L*, *I* system

$$\begin{array}{c} \dot{L}\left(t\right)=\beta \frac{S(t)(I(t)+fL(t))}{N(t)}-\left(\mu +\varphi \right)L\left(t\right)\\ \dot{I}\left(t\right)=\omega \varphi L\left(t\right)-\left(\alpha +\mu +\mu_{I}\right)I\left(t\right) \end{array}$$

yielding

$$\begin{array}{c} J=(\begin{array}{cc} f\beta -\varphi -\mu & \beta \\ \omega \varphi & -\alpha -\mu -\mu_{I} \end{array}) \end{array}$$ (5)

The characteristic equation of Eq (5) yields

$$\begin{array}{c} \xi^{2}+\xi \left(\varphi +2\mu +\alpha +\mu_{I}-f\beta \right)+\left(\alpha +\mu +\mu_{I}\right)\left(\varphi +\mu -f\beta \right)-\beta \omega \varphi . \end{array}$$

A simple calculation shows that a necessary and sufficient condition for Re ξ_1,2_ < 0 is

$$\begin{array}{c} \left(\alpha +\mu +\mu_{I}\right)\left(\varphi +\mu -f\beta \right)-\beta \omega \varphi >0 \end{array}$$

which is equivalent to

$$\begin{array}{c} \frac{\beta (\omega \varphi +f\left(\alpha +\mu +\mu_{I}\right))}{\left(\alpha +\mu +\mu_{I}\right)\left(\varphi +\mu \right)}<1. \end{array}$$

Setting

$$\begin{array}{c} R_{0}≔\frac{\beta (\omega \varphi +f\left(\alpha +\mu +\mu_{I}\right))}{\left(\alpha +\mu +\mu_{I}\right)\left(\varphi +\mu \right)} \end{array}$$

we find that the disease free equilibrium is locally asymptotically stable iff *R*_0_ < 1, and *R*_0_ being the reproductive rate.
